# Minimally Invasive Mitrofanoff in Children: Versatile Laparoscopic Strategies—From Low-Resource to Non-Robotic High-Cost Settings in an Exploratory Case Series

**DOI:** 10.3390/jcm15051954

**Published:** 2026-03-04

**Authors:** Elisa Cerchia, Marta Serpentino, Viet Nguyen Duy, Lorenzo Cirigliano, Massimo Catti, Elena Ruggiero, Quang Thanh Nguyen, Paolo Caione, Simona Gerocarni Nappo

**Affiliations:** 1 Pediatric Urology Unit, Department of Public Health and Paediatric Sciences, Regina Margherita Children’s Hospital, University Hospital of Health and Science, 10122 Turin, Italy; 2 Pediatric Surgery Unit, Department of Women’s and Children’s Health, University of Padua, 35122 Padua, Italy; 3 Surgical Department, National Hospital of Pediatrics, Dong Da District, Hanoi 100000, Vietnam; 4 College of Health Sciences, VinUniversity, Hanoi 10000, Vietnam; 5 Independent Researcher, 00100 Rome, Italy

**Keywords:** Mitrofanoff procedure, laparoscopy, pediatric urology, continent urinary diversion, minimally invasive surgery, resource-limited settings

## Abstract

**Background/Objectives**: The Mitrofanoff appendicovesicostomy (MAV) is the gold standard for creating a continent catheterizable channel in children unable to perform clean intermittent catheterization (CIC) through the native urethra. Minimally invasive surgery has progressively replaced open techniques in pediatric urology, offering improved recovery and favorable cosmetic outcomes, and robotic-assisted Mitrofanoff has gained popularity in recent years. However, the high costs and limited availability of robotic systems create disparities in access to pediatric urologic reconstruction, particularly in low- and middle-income countries. In this context, the laparoscopic Mitrofanoff (MAV-L) and the laparoscopic-assisted Mitrofanoff (MAV-LA) represent practical, cost-effective alternatives, valuable in institutions without robotic platforms or in resource-limited settings. Recent evidence demonstrates that advanced laparoscopic approaches remain feasible even for complex urological procedures, supporting their continued relevance in the robotic era. **Methods**: We conducted a retrospective case series including seven male children (aged 9–12 years) who underwent MAV between 2018 and 2023. Peri-operative data included demographics, operative time, length of hospitalization, and complications. Functional and aesthetic outcomes were assessed during long-term follow-up. Statistical analysis accounted for the small sample size by using non-parametric tests where appropriate. **Results**: Three patients (43%) underwent MAV-L and four (57%) MAV-LA. Mean operative time appeared longer in MAV-L (273.3 ± 20.5 min) than in MAV-LA (203.8 ± 24.3 min; exploratory *p* = 0.019). Hospital stay was 9 ± 0.8 days vs. 7.5 ± 0.5 days (*p* = 0.026). During follow-up (43.3 ± 10.9 vs. 26.3 ± 5.4 months; *p* = 0.034), two complications occurred, both in the MAV-L group (stomal stenosis and channel leakage). All patients reported excellent continence, ease of catheterization, and high cosmetic satisfaction. **Conclusions**: Both laparoscopic and laparoscopic-assisted Mitrofanoff techniques are safe, feasible, and effective in children. Favorable cosmetic satisfaction was reported in the fully laparoscopic subgroup, based on subjective assessment. Importantly, these laparoscopic techniques are sustainable alternatives to robotic surgery, offering accessibility and high-quality reconstructive care even in centers with limited financial and technological resources.

## 1. Introduction

The Mitrofanoff principle, first described in 1980 [1], introduced the concept of a continent catheterizable channel connecting the bladder to the abdominal wall using the appendix. This innovation revolutionized the management of pediatric patients unable to perform clean intermittent catheterization (CIC) via the native urethra, such as those with neurogenic bladder, posterior urethral valves, bladder exstrophy-epispadias complex (EEC) and complex urethral anomalies [2,3]. The procedure enables self-catheterization, reduces urinary tract infections, improves continence, allows preservation of renal function, and promotes independence and quality of life, representing a cornerstone of reconstructive pediatric urology [4].

The surgical technique involves the creation of a cutaneous stoma and a submucosal tunnel of adequate length within the bladder wall, in which the appendix is embedded, thus guaranteeing an effective continence mechanism. The gradual increase in intravesical pressure during bladder filling results in occlusion of the appendiceal lumen, ensuring continence. Consequently, this surgical procedure enables continence during bladder filling and bladder emptying at low pressure through CIC.

Over the past two decades, the field of pediatric urology has progressively shifted toward minimally invasive approaches, reflecting global trends [5,6]. The main steps of the minimally invasive procedure include harvesting of the appendix, creation of the appendicovesical anastomosis with an efficient anti-reflux mechanism, and construction of a cosmetic cutaneous stoma, either at the umbilicus or in the right iliac fossa. Laparoscopic and, more recently, robotic Mitrofanoff appendicovesicostomy (MAV) have demonstrated comparable functional results to open surgery, while offering reduced postoperative pain, shorter hospital stay, and superior cosmetic outcomes [2,4].

However, the adoption of robotic surgery remains uneven worldwide due to high equipment costs, maintenance expenses, and the need for specialized training [6]. This has created a disparity between high-income and low- to middle-income countries, where robotic systems are often unavailable and access to advanced pediatric urologic reconstruction remains limited [5]. In this context, laparoscopic Mitrofanoff appendicovesicostomy (MAV-L) represents a valuable and cost-effective alternative that can be performed using standard laparoscopic equipment, achieving the benefits of minimal invasiveness without the financial burden of robotic technology [2,5]. Furthermore, the laparoscopic-assisted Mitrofanoff (MAV-LA) has emerged as an intermediate option, simplifying intracorporeal suturing while maintaining small incisions and excellent visualization [4]. Both approaches may therefore expand access to high-quality reconstructive care, particularly in institutions with limited resources [5].

Despite increasing adoption, comparative data between MAV-L and MAV-LA in the pediatric population remain limited, and available studies are often heterogeneous with respect to age, underlying pathology, and associated procedures [2,4,6]. As a result, evidence regarding differences in operative time, peri-operative morbidity, and medium- to long-term functional outcomes is still scarce. Even small, well-characterized series may therefore provide clinically relevant information when surgical techniques and patient selection are homogeneous.

The aim of this retrospective case series was to describe peri-operative and functional outcomes of minimally invasive Mitrofanoff procedures, with exploratory subgroup analysis of fully laparoscopic (MAV-L) and laparoscopic-assisted (MAV-LA) techniques. In addition, we sought to contextualize these findings within the broader discussion on the role of laparoscopy as a sustainable and globally accessible alternative to robotic surgery, particularly in resource-limited settings.

## 2. Materials and Methods

### 2.1. Study Design and Population

This retrospective observational case series included all pediatric patients who underwent minimally invasive Mitrofanoff appendicovesicostomy (MAV) between January 2018 and December 2023. All consecutive patients meeting inclusion criteria were considered, and no eligible patient was excluded due to missing data. Surgical management was performed within a collaborative framework involving the Pediatric Urology team of Regina Margherita Children’s Hospital, University of Turin, and the Department of Pediatric Surgery of Hanoi. Seven male patients aged 9–12 years were included. Three patients underwent a totally laparoscopic Mitrofanoff procedure (MAV-L), and four underwent a laparoscopic-assisted Mitrofanoff (MAV-LA).

### 2.2. Inclusion and Exclusion Criteria

Inclusion criteria: children < 18 years requiring a continent catheterizable channel due to inability to perform clean intermittent catheterization (CIC) through the native urethra, with complete preoperative evaluation including blood tests, urinary tract ultrasonography, voiding cystourethrography (VCUG), and invasive urodynamic or videourodynamic assessment. Invasive urodynamic assessment was reported according to the terminology of the International Children’s Continence Society (ICCS) [7]. All patients demonstrated a pathological voiding phase with evidence of bladder outlet obstruction (BOO) or dysfunctional voiding (DV) with detrusor-sphincter dyssynergia, and a filling phase characterized by normal bladder capacity and compliance for age. Overactive bladder adequately controlled with anticholinergic therapy was not considered a contraindication.

Exclusion criteria: patients with low bladder compliance and/or overactive bladder requiring concomitant bladder augmentation, those requiring simultaneous bladder neck surgery, redo procedures, or incomplete clinical data were excluded. Although some patients showed detrusor overactivity, all had preserved bladder capacity and compliance and therefore did not require concomitant bladder augmentation.

### 2.3. Surgical Procedures

**Figure 1:**
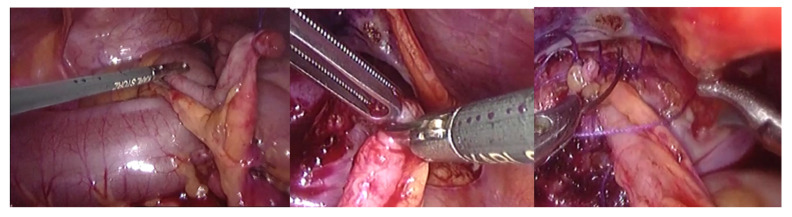
Laparoscopic Mitrofanoff (MAV-L) procedure. Intraoperative view showing the mobilization of the appendix and creation of the appendicovesical anastomosis.

MAV-L procedure: Patients were placed supine with slight Trendelenburg tilt. A Foley catheter was inserted to control bladder filling. Procedures were performed using three 5 mm trocars, with a fourth added if required. The appendix was evaluated for length and mobility; if inadequate, conversion to an open Monti channel was planned. Appendiceal mobilization preserved vascular pedicle integrity. The anterior bladder wall was prepared via a detrusor incision, and the appendix was anastomosed to the bladder mucosa using interrupted 5/0 polyglactin sutures. The appendix was embedded in a submucosal tunnel following the Lich-Gregoir anti-reflux principle. The bladder was fixed to the abdominal wall to enhance continence, and the appendix exteriorized through the umbilicus. The stoma was matured over a 12-Ch catheter left in place for 3 weeks (Figure 1).

**Figure 2:**
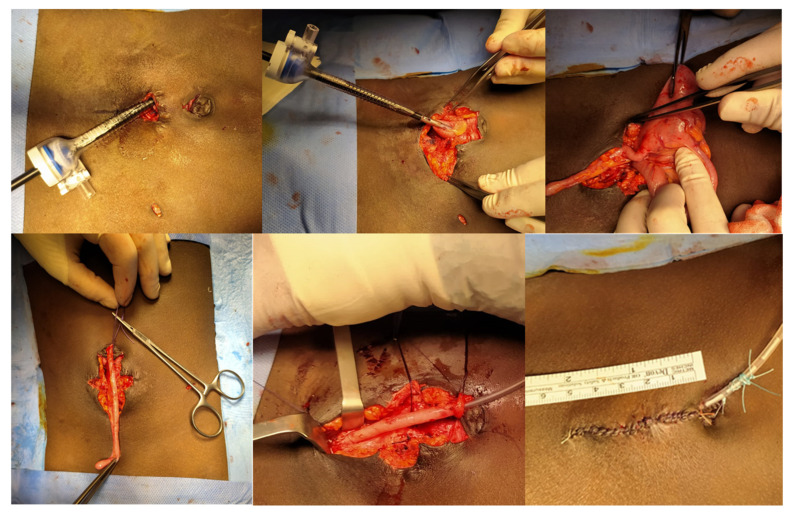
Laparoscopic-assisted Mitrofanoff (MAV-LA) procedure. Open extracorporeal phase showing appendicovesical anastomosis and umbilical stoma placement.

MAV-LA procedure: Patients were positioned supine with slight left rotation. Three 5 mm trocars were usually sufficient. The appendix was separated laparoscopically; in some cases, a small infra-umbilical incision allowed extracorporeal completion of the appendicovesical anastomosis, following the same principles as MAV-L (Figure 2).

All procedures were performed by the same experienced surgical team (SGN, PC). At the time of the study, all surgeons involved had more than 10 years of advanced laparoscopic experience in pediatric urology, including complex reconstructive procedures. This level of expertise allowed safe adoption of a fully laparoscopic Mitrofanoff approach, which is recognized as technically demanding.

### 2.4. Postoperative Care and Follow-Up

A 12-Ch Nelaton catheter was maintained for 3 weeks. Oral oxybutynin was administered during the same period to prevent bladder spasms. Bladder drainage was ensured via transurethral or suprapubic catheter until CIC was initiated. Oral intake and mobilization resumed within 48 h. Follow-up included physical examination, ultrasonography, and assessment of Mitrofanoff patency and continence at 1, 3, 6, and 12 months, then annually. Continence and cosmetic satisfaction were assessed subjectively by patients and caregivers, as no validated scoring system was applied.

### 2.5. Variables and Statistical Analysis

Collected variables included demographics, underlying diagnosis, operative time, length of hospital stay, complications (graded according to Clavien-Dindo), and long-term channel function. Due to the small sample size, all statistical analyses were considered exploratory and descriptive. Continuous variables are reported as mean ± standard deviation (SD) and categorical variables as counts and percentages. Exploratory subgroup comparisons were performed using non-parametric tests (Mann–Whitney U for continuous variables and Fisher’s exact test for categorical variables), with *p*-values reported for descriptive purposes only. All analyses were conducted using SPSS version 27 (IBM Corp., Armonk, NY, USA).

This study follows STROBE guidelines for retrospective observational studies.

## 3. Results

Seven male children (mean age 10.42 ± 0.95 years, range 9–12) underwent MAV-L (n = 3, 43%) or MAV-LA (n = 4, 57%). Mean weight was 29.67 ± 3.68 kg vs. 33.25 ± 11.60 kg. Statistical comparisons were performed using the Mann–Whitney U test due to the small sample size, and are considered exploratory.

### 3.1. Primary Diseases and Indications

Primary diagnoses included epispadias (n = 1), ureterocele with a solitary kidney (n = 1), urethral syringocele with a solitary kidney (n = 1), occult spinal dysraphism with anorectal malformation (n = 1), and posterior urethral valves (n = 3). CIC through the native urethra was impossible due to a sensitive urethra (n = 3) or anatomical obstacles (n = 3). One child required overnight bladder drainage because of polyuria and renal impairment. All patients demonstrated normal bladder compliance and capacity on urodynamic evaluation (Table 1).

### 3.2. Peri-Operative Outcomes

- Mean operative time: 273.33 ± 20.55 min (MAV-L, range 200–350) vs. 203.75 ± 24.33 min (MAV-LA, range 180–235), Mann–Whitney U *p* = 0.071, exploratory.
- Hospitalization appeared shorter in MAV-LA (exploratory *p* = 0.048), although the small sample size precludes definitive conclusions.
- Intra-operative complications: None in either group.

Due to the small sample size, these results are presented descriptively, and no definitive conclusions about superiority or inferiority of either technique can be drawn (Table 2).

### 3.3. Follow-Up and Complications

- **Mean follow-up:** 43.33 ± 10.87 months (MAV-L, range 32–58) vs. 26.25 ± 5.40 months (MAV-LA, range 20–33), Mann–Whitney U *p* = 0.060, exploratory.
- **Complications:** Two complications were observed in the MAV-L subgroup, while none were recorded in the MAV-LA subgroup; given the unequal follow-up and small sample size, these findings are descriptive.

The shorter follow-up in the MAV-LA group may underestimate late complications; therefore, comparisons between groups should be interpreted with caution (Table 3).

### 3.4. Functional and Cosmetic Outcomes

All patients and families reported excellent continence and ease of catheterization. Cosmetic and functional satisfaction were subjectively reported as excellent by all patients and families; it should be noted that aesthetic satisfaction and ease of catheterization were evaluated subjectively and were not assessed using validated questionnaires or standardized scores. Although one patient experienced stomal leakage requiring surgical revision, continence outcomes were assessed at the last follow-up, after resolution of complications. At the last follow-up, after management of postoperative complications, all patients reported absence of urinary leakage between catheterizations.

## 4. Discussion

The management of children with bladder dysfunction and a non-catheterizable urethra remains challenging. In the contemporary era of minimally invasive surgery, the rapid expansion of robotic platforms has led to the widespread assumption that conventional laparoscopy would progressively lose its role in complex urologic reconstruction. However, growing evidence suggests that laparoscopy remains a feasible, effective, and sustainable alternative for high-complexity procedures, particularly in settings where robotic systems are unavailable or impractical. Andras et al. highlighted that advanced laparoscopic techniques can achieve acceptable operative times and complication rates even for highly demanding urologic surgeries, emphasizing the importance of preserving laparoscopic expertise in the era of robotics [8].

In cases requiring bladder augmentation, continent catheterizable channels such as Mitrofanoff appendicovesicostomy or Monti channels are typically performed via an open approach in combination with augmentation. In contrast, in selected children with voiding dysfunction and preserved bladder capacity and compliance, Mitrofanoff appendicovesicostomy alone may represent the only necessary surgical intervention. Despite its potential advantages, reports on minimally invasive laparoscopic Mitrofanoff appendicovesicostomy (MAV-L) remain limited, likely reflecting both the technical complexity of the procedure and the increasing adoption of robotic-assisted platforms, which may have limited the dissemination of conventional laparoscopic expertise [2,6].

Previous studies have described both fully laparoscopic and hybrid (laparoscopic-assisted, MAV-LA) approaches for the creation of continent catheterizable channels, occasionally combined with other procedures such as bladder augmentation, nephrectomy, or antegrade continence enema placement [3,9]. Early contributions include the report by Strand et al., who described a transperitoneal laparoscopic nephrectomy combined with the creation of a catheterizable cutaneous ureterovesicostomy, applying the Mitrofanoff principle in an innovative manner [10]. The first fully laparoscopic Mitrofanoff appendicovesicostomy in a pediatric patient was subsequently reported by Hsu and Shortliffe [11]. Hybrid techniques were later introduced to simplify technically demanding steps; for example, Van Savage and Slaughenhoupt described laparoscopic mobilization of the appendix and right hemicolon, followed by completion of the appendicovesical anastomosis through a Pfannenstiel incision [2].

A key advantage of minimally invasive approaches is the possibility of early intraoperative assessment of the appendix. When the appendix is short, fibrotic, or unsuitable, alternative conduits such as retubularized ileal or sigmoid segments can be promptly considered [3,12,13]. Transverse retubularized sigmoid vesicostomy has been successfully used in pediatric patients lacking an adequate appendix, providing a reliable alternative conduit [12]. Early series on pure MAV-L demonstrated relatively short operative times, minimal blood loss, and absence of major complications, supporting the feasibility of the procedure [2,13,14]. Constructing a reliable continence mechanism remains one of the main technical challenges of MAV-L, regardless of whether the appendix is implanted on the posterior or anterior bladder wall. Posterior implantation has been favored by several authors, as it may provide a longer submucosal tunnel and a more effective flap-valve mechanism [3,4]. However, evidence supporting its superiority remains inconclusive.

Technical modifications, such as the U-Stitch technique, have further reduced operative time and simplified the appendicovesical anastomosis while maintaining satisfactory functional outcomes [9,10,11,12,13,14]. Anterior implantation is technically simpler but has been associated with urinary tract infections and stone formation, possibly related to less efficient bladder emptying [9]. Additionally, creation of the so-called hammock between the bladder and the anterior abdominal wall requires precise dissection within a restricted working space and may contribute to channel dysfunction when performed laparoscopically [3,9]. In this context, the MAV-LA approach may mitigate some of these difficulties, which is also suggested by shorter operative times and less technically demanding steps in our cohort.

In patients with complex neuro-urological conditions, such as spina bifida, MAV-L has shown significant improvement in patient satisfaction with clean intermittent catheterization, low blood loss, and no conversions to open surgery [9]. However, in children with high-pressure or severely dysfunctional bladders, complications such as urinary leaks and the need for revision surgery are more frequent [15]. The most frequent complications remain stomal leaks and stenosis, while severe events, such as appendiceal ischemia or the need for bladder augmentation, are rare.

In our series, no full conversions occurred among patients undergoing MAV-L. Postoperative morbidity appeared similar between subgroups, although the study is underpowered for formal comparison, with minor complications limited to one stomal stenosis and one stomal leak. These findings are consistent with published pediatric series, emphasizing that minimally invasive approaches are feasible and safe when performed by experienced teams.

Given the small sample size (n = 7), statistical comparisons between groups are exploratory and descriptive. Observed differences, such as shorter operative time in MAV-LA, should be interpreted as trends rather than definitive evidence of superiority. Similarly, the reported differences in follow-up duration between groups limit direct comparison of complication rates.

In our series, MAV-LA was associated with shorter operative time, representing an observed trend rather than definitive evidence, and facilitation of technically demanding steps, such as the appendicovesical anastomosis and submucosal tunneling [4,13,16]. In children with prior abdominal surgery, MAV-LA has been demonstrated to be safe and feasible, with operative times and complication rates comparable to those without previous surgery [17].

Minimally invasive techniques also carry specific intra-abdominal risks. Gander et al. reported an internal hernia over the mesoappendix following posterior MAV-L, emphasizing the importance of meticulous mesenteric handling and careful port placement [4]. A strength of the present study is the relatively long follow-up, exceeding three years in the MAV-L group. Long-term evaluation confirmed stable continence and sustained channel patency, consistent with previously reported pediatric series, including the long-term data provided by Weis et al. [9,13,16].

Overall, both MAV-L and MAV-LA appear to be safe and effective options for children requiring continent catheterizable channels. MAV-LA offers practical advantages, including reduced operative time and facilitation of technically demanding steps, while the minimally invasive setting allows early conduit assessment and substitution when necessary [4,17]. These findings support the adoption of minimally invasive Mitrofanoff techniques in specialized centers, while underscoring the importance of maintaining advanced laparoscopic expertise (Table 4).

### 4.1. Role of Laparoscopy in Resource-Limited Settings

In recent years, the rapid expansion of robotic surgery has largely overshadowed conventional laparoscopy; however, despite the well-recognized advantages of robotic platforms, laparoscopic and laparoscopic-assisted approaches remain essential in selected pediatric urologic scenarios. They not only provide effective alternatives but also allow less-experienced surgeons to develop the necessary technical skills more rapidly [18,19]. While robotic technology has undeniably advanced pediatric urologic reconstruction, its availability remains largely confined to well-funded tertiary centers. Acquisition costs of a robotic platform often exceed one million dollars, with substantial annual maintenance fees and dedicated training requirements, limiting accessibility in many pediatric hospitals worldwide [20,21].

This technological disparity has created a substantial gap between high-income countries and low- to middle-income countries (LMICs), where the implementation of robotic surgery is frequently unrealistic. In these settings, conventional laparoscopy often remains the only feasible minimally invasive option, underscoring the necessity for sustained expertise in non-robotic techniques. In this context, laparoscopic Mitrofanoff (MAV-L) and laparoscopic-assisted Mitrofanoff (MAV-LA) represent viable and sustainable alternatives. These approaches require only standard laparoscopic equipment, widely available even in peripheral or resource-constrained institutions [21,22]. The hybrid MAV-LA technique reduces the need for complex intracorporeal suturing, effectively shortening operative time and the learning curve without compromising outcomes. As a result, both approaches empower pediatric surgeons to perform advanced reconstructive procedures safely and effectively, regardless of robotic availability or institutional resources.

Several reports from Europe, Asia, and Africa have demonstrated that laparoscopic Mitrofanoff procedures can achieve outcomes comparable to robotic approaches when performed by experienced teams, with long-term patency, continence rates, and patient satisfaction similar to robotic cohorts [3,12,22,23,24]. Beyond clinical performance, laparoscopy offers clear advantages in cost-effectiveness, scalability, and broader dissemination of surgical skills. The recent availability of low-cost portable laparoscopic towers and reusable instruments further reinforces its feasibility in resource-limited environments [18,21].

Our series supports these observations. Despite a small cohort, perioperative and functional outcomes of MAV-L and MAV-LA compare favorably with published pediatric robotic-assisted Mitrofanoff series. Observed trends include longer operative time in MAV-L and slightly longer follow-up, but complication rates were low (one stomal stenosis, one stoma leak), and all patients reported excellent continence and satisfaction. It is important to underline that the surgeons involved in the present series had long-standing laparoscopic experience (>10 years), which allowed them to safely perform a fully laparoscopic Mitrofanoff in selected patients. However, the shorter operative times observed in the laparoscopic-assisted (MAV-LA) group suggest that the hybrid approach may represent a more reproducible and scalable technique. By reducing the need for complex intracorporeal suturing while preserving the benefits of minimally invasive surgery, MAV-LA may be more easily adopted by surgeons with basic or intermediate laparoscopic experience. This aspect is particularly relevant for low- and middle-income countries, where access to advanced robotic platforms is limited and where the dissemination of complex reconstructive procedures depends on feasibility, training requirements, and operative efficiency.

These findings suggest that minimally invasive laparoscopic approaches can achieve outcomes comparable to robotic techniques while remaining feasible in diverse resource settings (Table 5).

### 4.2. Limitations

The main limitations of this study include its retrospective design, small sample size, and single-center experience. Additionally, differences in follow-up duration and heterogeneity of underlying diagnoses limit the ability to draw definitive comparative conclusions between MAV-L and MAV-LA. Nevertheless, the uniformity of surgical technique and the long follow-up period strengthen the reliability of the findings.

Finally, this study should be interpreted as a case series with exploratory statistical analysis rather than a powered comparative trial, and observed differences between techniques should be considered descriptive trends rather than statistically definitive outcomes.

## 5. Conclusions

In this retrospective case series, both laparoscopic and laparoscopic-assisted Mitrofanoff techniques were feasible and safe minimally invasive options for continent urinary diversion in children. Favorable cosmetic satisfaction was reported in both subgroups, based on subjective assessment, whereas the laparoscopic-assisted variant reduces operative time and technical difficulty, with comparable functional outcomes in this small cohort. Importantly, our experience highlights that minimally invasive reconstructive surgery can be performed safely even in settings with limited resources. Laparoscopic Mitrofanoff procedures combine the advantages of minimal invasiveness with affordability and reproducibility, making them a viable option in both high- and low-resource institutions. Given the small sample size and retrospective design, these findings are exploratory, and larger prospective multicenter studies are needed to assess long-term outcomes, learning curves, cost-effectiveness, and quality-of-life measures. Preserving and disseminating laparoscopic expertise remains essential to ensure equitable access to pediatric urologic reconstruction worldwide. In this context, conventional laparoscopy represents a sustainable alternative to robotic-assisted techniques, bridging the gap in access to minimally invasive reconstructive care for children across diverse healthcare settings.

## Figures and Tables

**Table 1:** Demographic characteristics, indications, surgical details, and outcomes of patients undergoing Mitrofanoff appendicovesicostomy.

Pt	Age (Years)	Sex	Weight (kg)	Primary Diagnosis	Reason for Failed Urethral CIC	Preoperative Bladder Function *	Procedure (Year)	Operative Time (min)	Length of Stay (Days)	Complications
1	11	M	34	Ureterocele, solitary kidney	Posterior urethral diverticulum	Normal compliance; BOO	MAV-L (2018)	300	9	Stomal stenosis
2	10	M	30	Urethral syringocele, solitary kidney	Urethral obstruction	OAB controlled with anticholinergic therapy; normal compliance; BOO	MAV-L (2021)	250	10	Stomal leakage
3	9	M	25	Posterior urethral valves	Painful/sensitive urethra	Normal compliance; BOO	MAV-L (2020)	270	8	None
4	12	M	53	Spinal dysraphism, ARM, transplanted kidney	Painful/sensitive urethra	Increased compliance; active VUR; DV with high voiding pressure (200 cm H_2_O)	MAV-LA (2021)	235	8	None
5	11	M	26	Epispadias	Urethral stenosis	Normal compliance; BOO	MAV-LA (2021)	220	7	None
6	10	M	24	Posterior urethral valves	Painful/sensitive urethra	Normal compliance; BOO	MAV-LA (2022)	180	8	None
7	10	M	30	Posterior urethral valves	Polyuria; recurrent UTI during transurethral CIC	Normal compliance; active VUR; BOO	MAV-LA (2022)	180	7	None

Abbreviations: CIC = clean intermittent catheterization; MAV-L = laparoscopic Mitrofanoff appendicovesicostomy; MAV-LA = laparoscopic-assisted Mitrofanoff appendicovesicostomy; OAB = overactive bladder; BOO = bladder outlet obstruction; DV = dysfunctional voiding; VUR = vesicoureteral reflux; ARM = anorectal malformation. * All patients had normal bladder compliance and capacity for age and did not require concomitant bladder augmentation.

**Table 2:** Demographic and peri-operative characteristics.

Variable	MAV-L (n = 3)	MAV-LA (n = 4)	*p*-Value *
Age (years)	10.42 ± 0.95	10.25 ± 0.96	0.86
Weight (kg)	29.67 ± 3.68	33.25 ± 11.60	0.62
Operative time (min)	273.33 ± 20.55	203.75 ± 24.33	0.071
Hospitalization (days)	9 ± 0.81	7.5 ± 0.50	0.048
Intra-operative complications	0	0	–

* Statistical comparisons for continuous variables performed using Mann–Whitney U test, exploratory due to small sample size.

**Table 3:** Post-operative outcomes and follow-up.

Variable	MAV-L (n = 3)	MAV-LA (n = 4)	*p*-Value
Follow-up, months (mean ± SD)	43.33 ± 10.87	26.25 ± 5.40	0.034
Patients with postoperative complications, n	2	0	0.14
Stomal stenosis	1	0	–
Channel leakage	1	0	–
Continence at last follow-up *	3/3	4/4	–
Ease of catheterization ^†^	Subjectively satisfactory in all patients	Subjectively satisfactory in all patients	–
Cosmetic satisfaction ^†^	Subjectively satisfactory in all patients	Subjectively satisfactory in all patients	–

* Continence was assessed at the last follow-up after management and resolution of postoperative complications and was defined as absence of urinary leakage between catheterizations, without pad use. ^†^ Ease of catheterization and cosmetic satisfaction were evaluated subjectively based on patient and family reports; no validated scoring system was used.

**Table 4:** Overview of pediatric minimally invasive Mitrofanoff procedures. Includes study, year, procedure type, number of patients (N), age, implant site (P = posterior, A = anterior), operative time, main complications, and follow-up. MAV-L = laparoscopic Mitrofanoff; MAV-LA = laparoscopic-assisted Mitrofanoff.

Study	Year	Procedure	N	Age (Years)	Implant Site	Operative Time (min)	Main Complications	Follow-Up (Months)
Nerli	2012	MAV-L	6	12.8 (9–16)	P	139.6	1 UTI	33
Badawy	2012	MAV-L	4	6 (3–9)	P/A	210	None	12.5
Weller	2012	MAV-L	6	10 (4–16)	A	183	1 Stomal leakage	6.8
Reddy	2015	MAV-L	11	11 ± 3.2	P	144	3 Stomal leakage, 3 stenosis, 4 urethral leak	34
Blanc	2014	MAV-L	15	9	P	255	5 urinary leakage, 3 conversions	18
Kim	2019	MAV-LA	34	6–9.9	A	255/267	6 stenosis, 2 stomal leakage	26
Gander	2022	MAV-L	15	8.8 ± 3.1	P	median 217.3 (140–300)	1 ileus, 1 internal hernia, 1 stenosis	21
Weis et al.	2024	MAV-L/Robotic	29	8 (6–13)	P	310	3 conversion, 9 stomal leakage	60
Our series	2024	MAV-L/MAV-LA	7	10.4 ± 0.9	A	273/203 ± 20.5/24.3	1 stenosis, 1 stomal leakage	43/26

Abbreviations: MAV-L = laparoscopic Mitrofanoff; MAV-LA = laparoscopic-assisted Mitrofanoff; P = posterior implantation; A = anterior implantation.

**Table 5:** Comparison of pediatric robotic and laparoscopic Mitrofanoff studies. Summary of patient numbers, age, operative time, length of stay, and reported complications or outcomes for laparoscopic (MAV-L/MAV-LA) and robotic-assisted (RALMA/RALIMA) approaches.

Study	N	Procedure	Operative Time (min)	Length of Stay (Days)	Complications
Nguyen et al., 2009	10	Robotic	323 median (181–507)	5	1 urinary leak, 2 minor incontinence
Famakinwa et al., 2013	18	Robotic	494 ± 108	6 ± 1.5	Stenosis, hernia, minor
Chan et al., 2015	1	Robotic	555	7	1 stomal stenosis
Gundeti et al., 2016	88	Robotic	424 ± 120	5.2 ± 2.8	29.5% early complications, 12.5% stomal revision
Chung et al., 2015	3	Robotic	165	4	1 stomal leak
Adamic et al., 2020	24 (20 RALI)	Robotic	573 ± 142	6 ± 1	35% early complications; long-term: 25% bladder stones, 15% upper tract stones, 5% bladder rupture, 5% SBO
Juul et al., 2021	17 (5 robotic, 12 open)	Robotic/Open	249/231	5–6	40% stenosis robotic vs. 25% open
**Our series**	7	MAV-L/MAV-LA	273/203 ± 20.5/24.3	9/7.5 ± 0.81/0.5	1 stenosis, 1 stomal leakage

Abbreviations: SBO = small bowel obstruction; RALI = robotic-assisted laparoscopic ileal conduit; MAV-L = laparoscopic Mitrofanoff; MAV-LA = laparoscopic-assisted Mitrofanoff.

## Data Availability

All relevant data supporting the findings of this study are included within the article. Additional anonymized data are available from the corresponding author upon reasonable request.
